# Comparison of apical transportation and centering ratio between Bassi Logic and Protaper-Next NiTi rotary systems in curved canal by cone beam computed tomography: an in vitro study

**DOI:** 10.1186/s12903-026-08992-2

**Published:** 2026-07-04

**Authors:** Maryam Khalili, Abbas Shokri, Hamed Karkehabadi, Fatemeh Ahanj, Amir-Masood Pour Mohammad, Mohammad Reza Moayedi

**Affiliations:** 1 Department of Endodontics, Faculty of Dentistry, Hamadan University of Medical Sciences, Hamadan, Iran; 2 Department of Oral and Maxillofacial Radiology, Faculty of Dentistry, Dental Research Center, Hamadan University of Medical Sciences, Hamadan, Iran; 3 Faculty of Dentistry, Dental Research Center, Hamadan University of Medical Sciences, Hamadan, Iran

**Keywords:** Canal Transportation, Centering Ratio, Rotary File Systems, ProTaper Next, Bassi Logic

## Abstract

**Background:**

Preservation of the original root canal anatomy is essential for the long-term success of endodontic treatment. Effective cleaning and shaping of the root canal system are critical steps in this process; however, canal transportation is a common procedural error during the preparation of curved canals. This study aimed to compare canal transportation and centering ability between two rotary file systems, ProTaper Next and Bassi Logic, in curved root canals using cone-beam computed tomography (CBCT).

**Methods:**

Forty extracted human mandibular first molars with mature apices and an apical curvature ranging from 10° to 30° were selected. These samples were randomly allocated into two groups of 20 teeth, ensuring similar average curvatures. Root canal preparation was performed using either the ProTaper Next or Bassi Logic rotary file systems, strictly adhering to the manufacturers’ protocols. CBCT images were acquired using a Kodak 9600 system (Care Stream, Paris, France), both before and after instrumentation. Canal transportation is a common procedural error during the preparation of curved canals. Statistical analysis was performed using an independent t-test to compare the two groups, with the significance level set at *P* < 0.05. Statistical analysis was performed using an independent t-test, with the significance level set at *P* < 0.05.

**Results:**

The ProTaper Next system demonstrated greater canal transportation and a lower centering ratio across all evaluated apical levels compared to the Bassi Logic system. Statistically significant differences in canal transportation were observed at the 3 mm level in the mesiodistal plane and at the 5 mm level in the buccolingual direction (*P* < 0.05). However, no statistically significant differences in centering ratio were found between the two systems at any of the evaluated levels (*P* > 0.05).

**Conclusions:**

Within the limitations of this in vitro study, both systems demonstrated acceptable shaping ability in curved canals; however, Bassi Logic showed less canal transportation at certain apical levels, which may support better preservation of canal anatomy in clinical practice.

## Background

Cleaning and shaping of the root canal system are essential steps for successful endodontic treatment, aiming to eliminate microorganisms while preserving the original canal anatomy [1]. Preparation of curved canals remains challenging and may lead to procedural errors such as canal transportation, ledge formation, and perforation, which can negatively affect treatment outcomes [2, 3]. Canal transportation occurs when instruments deviate from the original canal path due to their tendency to straighten within curved canals, potentially compromising cleaning efficiency and obturation quality [4, 5]. Excessive transportation in the apical third may jeopardize the apical seal and long-term prognosis of endodontic therapy [5].

Maintaining centering ability during instrumentation is therefore critical to preserve dentin thickness and prevent structural weakening of the root [6–8]. Nickel–titanium (NiTi) rotary systems have largely replaced stainless steel instruments because of their superior flexibility and shape memory properties, which allow safer preparation of curved canals with fewer iatrogenic errors [9–12]. The shaping performance of NiTi instruments is influenced by their metallurgical treatment, taper, cross-sectional design, and motion kinematics [7, 8, 11].

Accurate assessment of canal transportation and centering ability requires reliable three-dimensional imaging techniques. Cone-beam computed tomography (CBCT) has been widely used in endodontic research as a non-destructive and reproducible method for evaluating morphological changes before and after instrumentation. CBCT enables precise measurement of dentin removal and canal deviation at different apical levels, providing quantitative analysis of shaping performance [13, 14].

ProTaper Next (PTN) (Dentsply Maillefer, Switzerland) is a widely used multi-file rotary system manufactured from M-Wire alloy. This thermomechanically treated alloy enhances flexibility and cyclic fatigue resistance compared to conventional NiTi instruments [15–17]. Additionally, its off-center rectangular cross-section and progressive taper design are intended to reduce the contact area between the file and canal walls, potentially minimizing torsional stress and improving debris removal efficiency [15, 16]. Due to its widespread clinical use, extensive evaluation in contemporary endodontic studies, M-Wire alloy, variable taper design, and off-center rectangular cross-section, ProTaper Next is frequently used as a reference system in comparative shaping studies.

Bassi Logic (Bassi Endo, Brazil) is a heat-treated NiTi rotary system that incorporates dedicated glide path instruments with conservative tapers (0.01) followed by shaping files with greater tapers (0.03–0.05). The inclusion of specific glide path instruments within the system is intended to facilitate safer negotiation of curved canals, reduce stress on shaping files, and maintain canal curvature during subsequent enlargement [18–20]. Recent evidence suggests that glide path preparation significantly reduces canal transportation and improves centering ability of NiTi systems [10]. Furthermore, emerging studies evaluating Logic-type systems have reported favorable mechanical behavior and shaping performance in curved canals [21].

Despite these advancements, direct comparisons between Bassi Logic and established multi-file rotary systems such as ProTaper Next remain limited. Therefore, the present study aimed to compare canal transportation and centering ability between ProTaper Next and Bassi Logic systems in curved root canals using CBCT imaging.

## Methods

This study was conducted through in vitro laboratory tests at the Faculty of Dentistry, Hamedan University of Medical Sciences, during 2023–2024. This in vitro study was reviewed and approved by the Ethics Committee of Hamadan University of Medical Sciences (Approval No. IR.UMSHA.REC.1397.464). The research received approval from the Ethics Committee of Hamedan University of Medical Sciences (ethics code IR.UMSHA.REC.1397.464).

A total of 60 human mandibular first molars were initially screened. From these, 40 teeth that fulfilled the inclusion criteria (mesiodistal root curvature between 10° and 30° in the mesial root) were selected. The sample size was determined based on power analysis using G Power software, considering a significance level of 0.05 and a statistical power of 80%, according to previously published studies evaluating canal transportation and centering ability. Accordingly, 20 teeth per group were considered sufficient to detect clinically relevant differences between the two systems.

All teeth had been extracted due to periodontal disease or caries unrelated to this study and were examined under ×5 magnification to exclude cracks or fractures. The selected teeth were then randomly allocated into two groups (*n* = 20 each) using a computer-generated random number table.

The selected teeth were disinfected in a 1:10 dilution of 5% sodium hypochlorite (final concentration ≈ 0.5%) for a limited period and subsequently stored in normal saline until use. This low concentration is not expected to adversely affect dentin properties.

**Fig. 1:**
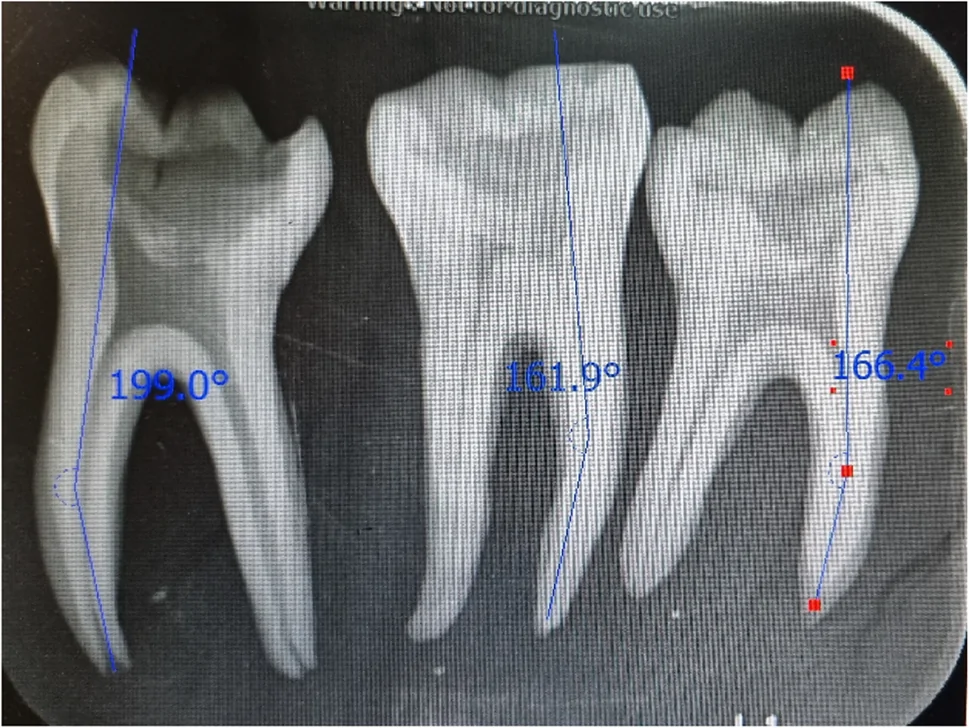
Periapical radiograph demonstrating root curvature determination of the samples

To accurately measure root curvature and select appropriate samples, periapical radiographs were taken using an intraoral digital imaging system (PSP Optime, Soredex, Tuusula, Finland) with a size 2 sensor and a Minray Soredex device (Tuusula, Finland) set at 60 kVp, 10 mA, and 0.2 s. In addition, CBCT scans were obtained for each sample, with a single tooth positioned at the center of the field of view (FOV) to minimize distortion and enhance image accuracy. Root curvature angles were measured according to the Schneider criterion using Scanora software (Soredex, Tuusula, Finland) (Fig. 1). All measurements were performed by a single calibrated examiner under standardized conditions to minimize measurement variability.

A line was drawn along the tooth’s long axis, and a second line extended from the apical foramen to the point where the canal first deviated from the long axis. The acute angle between these two lines defined the degree of canal curvature. Only teeth with mesiodistal curvature of 10–30° in the mesial root were included. Teeth with calcification, internal or external resorption, root fracture, severe or bidirectional curvature, or an open apex were excluded. The 40 selected teeth were equally divided into two groups of 20.

**Fig. 2:**
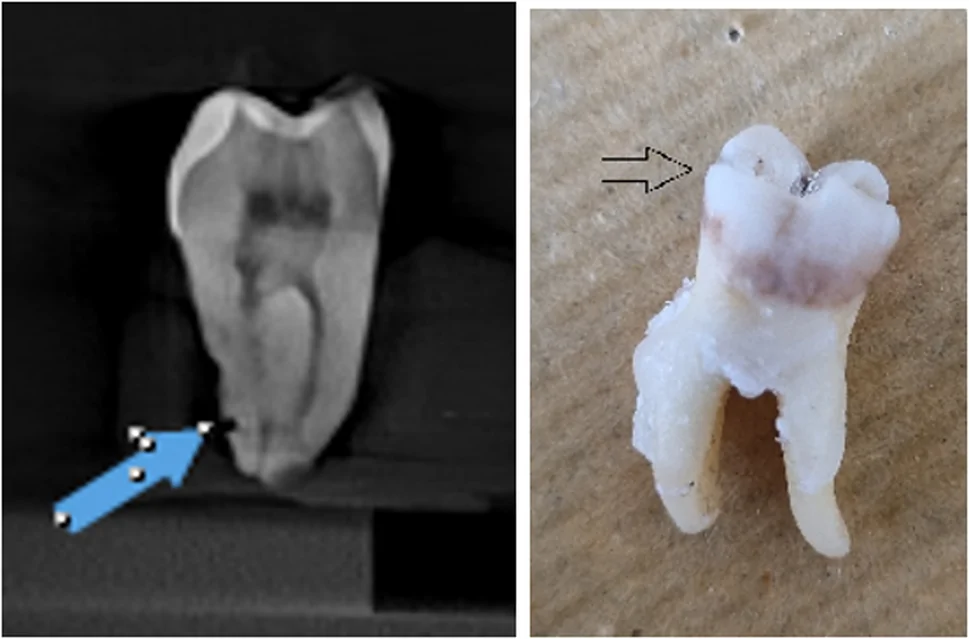
Illustrations of apical reference point shortening and mesial root grooving

Access cavities were prepared in all teeth using a high-speed round carbide bur (Dentsply, Maillefer, Ballaigues, Switzerland). Working length was established using a K-10 file (Mani, Japan) until it was 1 mm short of the apical foramen, measured from the occlusal reference point. To standardize, the occlusal reference was shortened to achieve a uniform working length of 19 mm for all samples (Fig. 2).

**Fig. 3:**
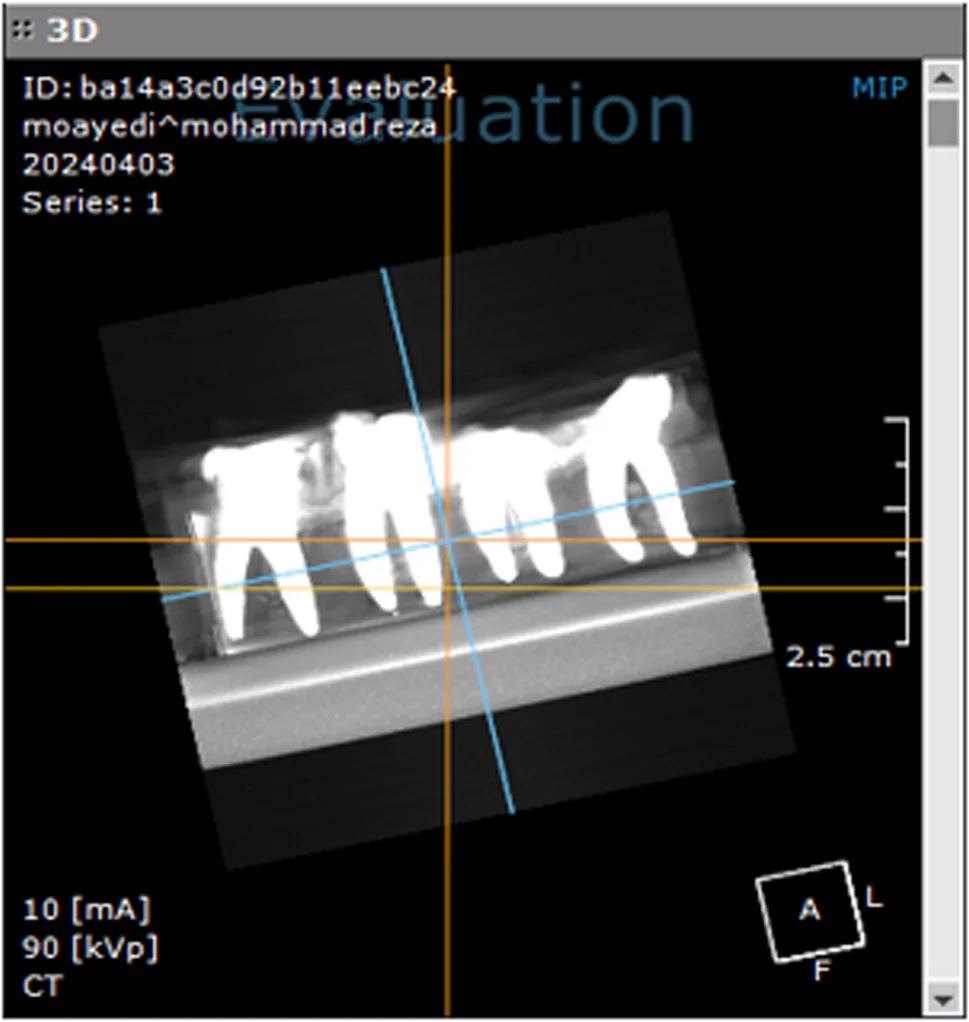
Sagittal section used for alignment of the acrylic block’s axis

Grooves were marked on the mesial root at 3, 4, and 5 mm from the apex. For consistent CBCT imaging and reproducibility, teeth were embedded in paraffin blocks, and a small piece of orthodontic wire was placed on one side of each block to facilitate scan orientation (Fig. 3). Prior to instrumentation, CBCT images were acquired using a Kodak 9600 unit (Carestream, Paris, France) with the following settings: 90 kVp, 10 mA, 12-second exposure time, 90-micron voxel size, and a 5 × 5 cm field of view in high-resolution mode.

Although multiple teeth were embedded in blocks for standardized positioning, each CBCT scan was performed with a single tooth positioned at the center of the field of view (FOV). This approach minimized potential magnification differences, geometric distortion, and beam hardening artifacts. All images were saved in DICOM format.

Mechanical preparation began with the creation of a glide path using a size 15 K-file. All instrumentation procedures were performed by a single experienced operator to eliminate inter-operator variability.

In the first group, canals were prepared using the ProTaper Next rotary system (Dentsply Sirona) operated by an X-Smart motor (Dentsply Maillefer, Ballaigues, Switzerland) at 300 rpm and 2 N·cm torque. The instrumentation sequence consisted of X1 (17/0.04), followed by X2 (25/0.06).

In the second group, canals were prepared using the Bassi Logic rotary system (Dentsply Maillefer, Ballaigues, Switzerland), operated with the same X-Smart motor at 350 rpm and 2 N·cm torque. The instrumentation sequence consisted of a size 15 file with a 3% taper (glide path file), followed by a size 25 file with a 6% taper (shaping file). The rotational speed and torque settings for each system were selected according to the manufacturers’ recommendations in order to reflect the intended clinical use of each rotary system.

In both groups, canals were irrigated with normal saline between the use of each file. Each file was used to prepare a maximum of four canals.

To simulate body temperature conditions, teeth were maintained in water at 37 ± 1 °C during instrumentation, and the temperature was continuously monitored using a thermometer.

This approach was adopted because the mechanical behavior and flexibility of thermally treated nickel-titanium instruments are temperature-dependent and may differ under simulated intraoral conditions.

Following preparation, CBCT images were reacquired under identical conditions to the initial scans.

Canal transportation and centering ratio were calculated using OnDemand 3D Dental software (Cybermed, Seoul, South Korea) for dentin thickness measurements. In the sagittal view, a reference line was drawn along the long axis of the canal curvature to ensure that cross-sectional slices were obtained perpendicular to the canal.

**Fig. 4:**
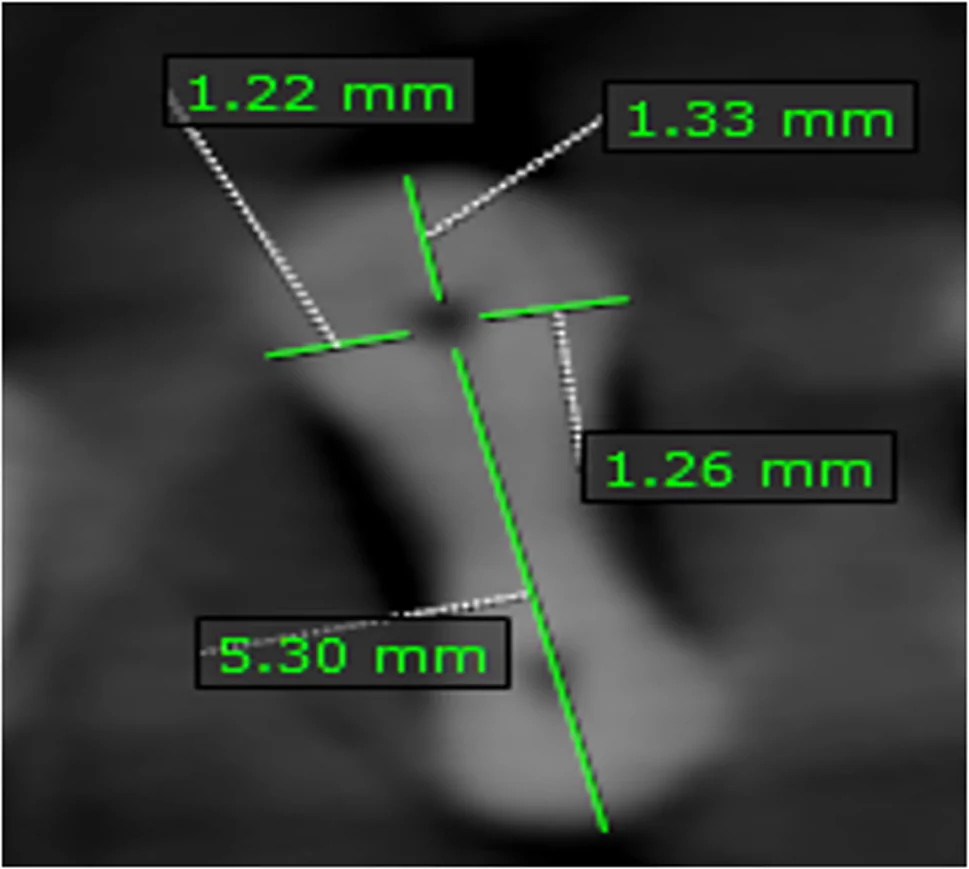
Detailed axial CBCT images illustrating dentin thickness measurements taken at the mesial, distal, buccal, and lingual aspects of the mesiobuccal root

In the axial view, measurements were performed at 3, 4, and 5 mm from the mesiobuccal apex, as these levels are more susceptible to procedural errors such as ledging, transportation, zipping, and perforation. At each level, dentin thickness was measured in millimeters at four points (mesial, distal, buccal, and lingual) using the ruler tool, with a slice thickness and interval of 0.5 mm (Fig. 4). The same measurement protocol was applied to both pre-instrumentation and post-instrumentation CBCT images under identical conditions.

The following variables were defined:

M1, D1, B1, and L1 represent the shortest distances from the mesial, distal, buccal, and lingual external root surfaces to the canal wall before instrumentation, respectively.

M2, D2, B2, and L2 represent the corresponding distances after instrumentation.

Canal transportation was calculated at each level using the following formulas:

$$\text{Mesiodistal transportation}={\mid\left(\mathrm{D2}-\mathrm{D2}\right)-\left(\mathrm{M2}-\mathrm{M1}\right)\mid}$$

$$\text{Buccolingual transportation}={\mid\left(\mathrm{L2}-\mathrm{L1}\right)-\left(\mathrm{B2}-\mathrm{B1}\right)\mid}$$

A value of 0 indicates no canal transportation.

The centering ratio was calculated as follows:

$$\text{Mesiodistal centering ratio}={\left(\mathrm{M2}-\mathrm{M1}\right)/\left(\mathrm{D2}-\mathrm{D1}\right)}\;\mathrm{or}\;\left(\mathrm{D2}-\mathrm{D1}\right)/\left(\mathrm{M2}-\mathrm{M1}\right)$$

$$\text{Buccolingual centering ratio}={\left(\mathrm{B2}-\mathrm{B1}\right)/\left(\mathrm{L2}-\mathrm{L1}\right)}\;\mathrm{or}\;\left(\mathrm{L2}-\mathrm{L1}\right)/\left(\mathrm{B2}-\mathrm{B1}\right)$$

In each case, the smaller value was divided by the larger one to obtain a value between 0 and 1. A value closer to 1 indicates better centering ability.

All measurements were independently performed by a maxillofacial radiologist and an endodontist, both experienced in using OnDemand 3D software. To assess intra-observer reliability, all CBCT images were re-evaluated after a one-month interval. Data were analyzed using SPSS software (version 22.0; IBM Corp., Armonk, NY, USA). An independent t-test was used to compare the two file systems, and one-way ANOVA was used for intragroup comparisons. A significance level of *P* < 0.05 was considered statistically significant.

## Results

This study involved 40 human mandibular first molars, all exhibiting similar root curvature (10 to 30 degrees) and healthy root structures. These teeth were randomly allocated into two groups of 20, ensuring standardized canal curvature across both. Following preparation with either the ProTaper Next or Bassi Logic system, we assessed the degree of apical canal transportation and the centering ratio of the mesiobuccal canal in each tooth. All statistical analyses were conducted with a significance level of 5% (*p*-value < 0.05). Table 1 presents the mean canal transportation, measured in millimeters, at 3, 4, and 5 mm from the apex for both the ProTaper Next and Bassi Logic systems.

**Table 1:** Comparison of canal transportation (mean ± SD, mm) between ProTaper Next and Bassi Logic systems at 3, 4, and 5 mm from the root apex in Mesiodistal (MD) and Buccolingual (BL) directions

system zone		3 mm	4 mm	5 mm
DCT-MD	ProTaper Next	0.085 ± 0.035	0.099 ± 0.024	0.091 ± 0.072
	Bassi Logic	0.065 ± 0.032	0.088 ± 0.027	0.085 ± 0.025
	*P*- value	0.035	0.060	0.251
DCT-BL	Protaper Next	0.110 ± 0.017	0.106 ± 0.013	0.145 ± 0.036
	Bassi Logic	0.096 ± 0.016	0.092 ± 0.017	0.099 ± 0.020
	*P* value	0.062	0.053	0.003

*DCT* Degree of canal transportation, *MD* Mesiodistal, *BL* Buccolingual. Values are presented as mean ± SD. *P*-values represent intergroup comparisons between ProTaper Next and Bassi Logic systems (independent t-test, significance level set at *p* < 0.05)

**Fig. 5:**
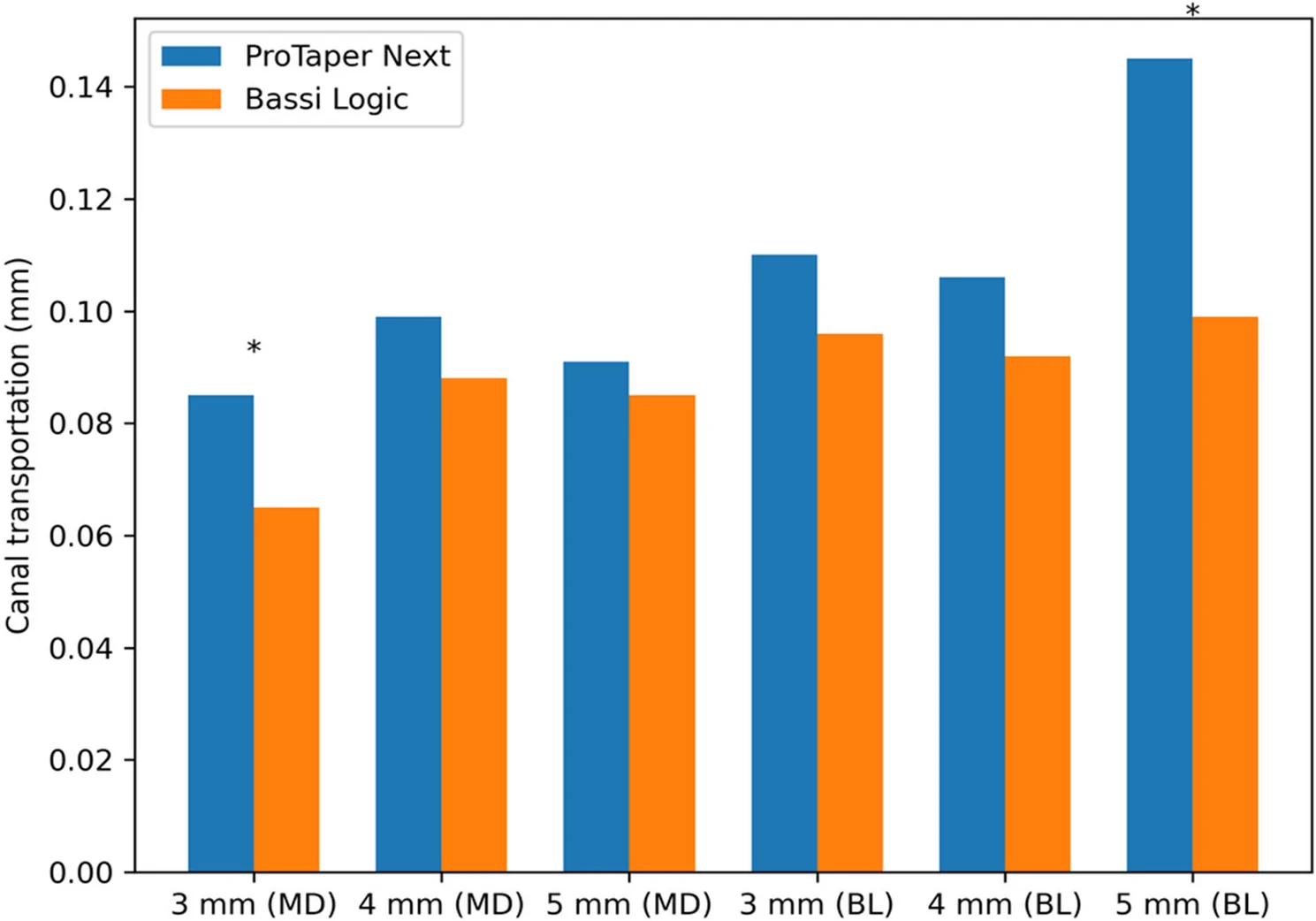
Canal transportation (mm) at 3, 4, and 5 mm from the root apex in Mesiodistal (MD) and Buccolingual (BL) directions for ProTaper Next and Bassi Logic systems. Statistically significant differences between groups are indicated by an asterisk (*)

Table 1’s findings reveal that, in the mesiodistal plane at 3 mm from the apex, the ProTaper Next system exhibited significantly greater mean canal transportation compared to the Bassi Logic system (*p* < 0.05). Similarly, in the buccolingual plane at 5 mm from the apex, the ProTaper Next system also showed significantly greater mean canal transportation than the Bassi Logic system (*p* < 0.05). While the ProTaper Next system consistently demonstrated greater mean transportation at other distances and planes, these differences were not statistically significant. Within each group, no statistically significant differences in transportation were observed at 3, 4, or 5 mm from the apex, regardless of whether measured in the mesiodistal or buccolingual planes (Fig. 5).

No statistically significant difference was observed in the mean canal centering ratio between the ProTaper Next and Bassi Logic systems at 3, 4, or 5 mm from the apex in either the mesiodistal or buccolingual planes (*p* > 0.05) (Table 2). Although the Bassi Logic system demonstrated slightly higher mean centering ratios, the differences were not statistically significant.

**Table 2:** Comparison of canal centering ratios (mean ± standard deviation) at 3, 4, and 5 mm from the root apex in mesiodistal and buccolingual directions for ProTaper Next and Bassi Logic systems

system zone		3 mm	4 mm	5 mm
CR-MD	Protaper Next	0.426 ± 0.433	0.578 ± 1.203	0.509 ± 0.021
	Bassi Logic	0.530 ± 0.032	0.635 ± 0.017	0.626 ± 0.013
	*p*-value	0.512	0.356	0.211
CR-BL	Protaper Next	0.212 ± 0.129	0.284 ± 0.065	0.234 ± 0.413
	Bassi Logic	0.333 ± 0.017	0.295 ± 0.012	0.248 ± 0.043
	*P* value	0.607	0.358	0.080

*CR* Centring Ratio, *MD* Mesio-distal, *BL* Bucco-Lingual

**Fig. 6:**
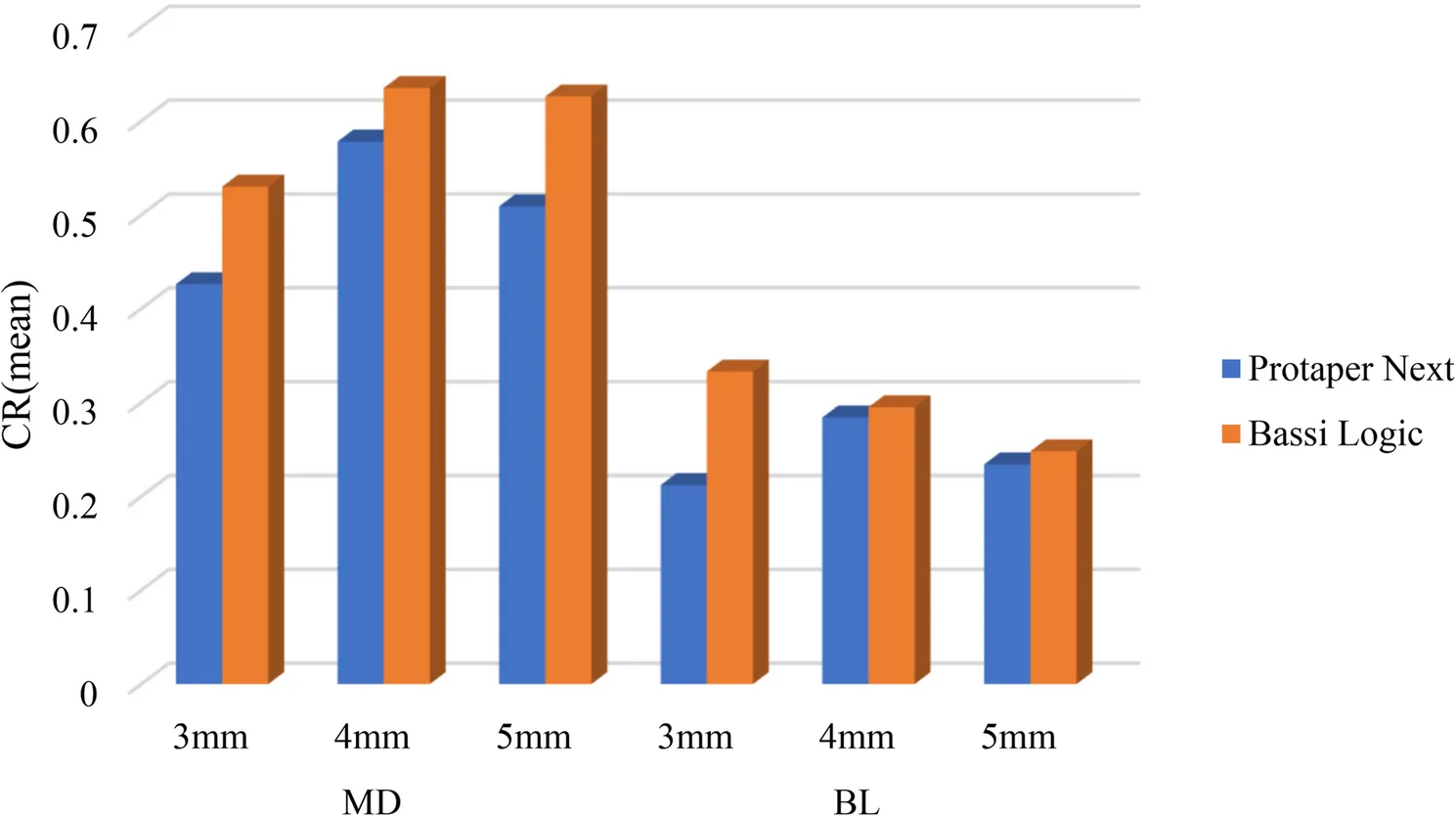
Comparison of canal centering ratios (mm) at 3, 4, and 5 mm from the apex in mesiodistal and buccolingual planes for ProTaper Next and Bassi Logic systems

Furthermore, intragroup comparisons revealed no statistically significant differences in the centering ratio at 3, 4, or 5 mm from the apex, for either the mesiodistal or buccolingual planes, within any of the groups (Fig. 6).

## Discussion

The present investigation aimed to assess canal transportation patterns and centering ratios when comparing ProTaper Next and Bassi Logic systems in curved root canals. CBCT imaging was selected as the preferred method due to its enhanced precision in identifying three-dimensional canal modifications compared to traditional radiographic techniques [1, 22].

The analysis revealed increased transportation levels with the ProTaper Next system, particularly at 3 mm (mesiodistal direction) and 5 mm (buccolingual direction) from the apex. At the same time, no statistically significant differences were observed in centering ratio measurements. These observations are consistent with previous research that examined ProTaper Next performance against other systems, including Reciproc Blue, WaveOne Gold, and Mtwo, highlighting how instrument design significantly influences shaping outcomes [2, 6].

The comparative evaluation demonstrated that canal transportation between these two rotary systems differed significantly in specific anatomical planes. In the mesiodistal plane at 3 mm from the apex and in the buccolingual plane at 5 mm from the apex, the ProTaper Next system exhibited substantially greater transportation compared to the Bassi Logic system.

Regarding centering ratio measurements in both mesiodistal and buccolingual planes, similar performance was observed between the two rotary systems at distances of 3, 4, and 5 millimeters from the tooth apex. While the Bassi Logic rotary system demonstrated a higher average centering ratio compared to ProTaper Next, this difference did not reach statistical significance.

The observed higher transportation degree with the ProTaper Next system aligns with findings from investigations comparing this system to WaveOne Gold, Twisted Files, Reciproc Blue, and Mtwo systems [2, 4, 6]. Similarly, the lower centering ratio demonstrated by the ProTaper Next system corresponds with research comparing it to One Shape, Reciproc Blue, and WaveOne Gold systems [3, 4]. The ProTaper Next system’s reduced ability to maintain central positioning may be attributed to its variable taper design along the cutting edge, contrasting with the Bassi Logic’s consistent taper throughout the instrument length. Research has established that instruments featuring constant taper in the apical region exhibit superior centering ratios compared to those with variable, progressive taper designs along the cutting edge [13].

The ProTaper Next file incorporates a rectangular cross-section with off-center rotation mechanics. Consequently, as the distance from the tip increases, dentin cutting is performed by the file’s outermost portion. Von der Weyer et al. documented that ProTaper Next files remove greater dentin volumes compared to alternative instruments. This phenomenon relates to multiple factors, including the “envelope of motion” effect, which can result in prepared canal tapers exceeding the original file specifications. Their study revealed that the final preparation taper with PTN reached 6.7%. In comparison, the coronal section of all PTN files features a 4% taper, demonstrating that the final preparation geometry differs from the original file shape [2].

The off-center rotation characteristic creates centrifugal force and envelope of motion effects, resulting in increased dentin removal, greater transportation, and reduced centering ratio. Additionally, the ProTaper Next file’s increased transportation may relate to its larger taper (exceeding 6% but remaining below 7%) and consequently larger cross-sectional area at 3–5 millimeters from the tip compared to the Bassi Logic file’s 6% taper. This dimensional difference may increase dentin removal and cause more pronounced canal modifications. The Bassi Logic file features a cross-sectional design similar to Reciproc Blue, incorporating a double helix configuration. A 2018 investigation by Priscila et al. comparing WaveOne Gold, ProTaper Next, and Reciproc Blue systems found no significant differences in transportation and centering capabilities. However, Reciproc Blue demonstrated superior performance compared to the other systems [6]. Given the cross-sectional similarity between Bassi Logic and Reciproc Blue, the reduced transportation and improved centering ratio of the Bassi Logic file may be attributed to its cross-sectional design.

A study by Pinheiro et al., which showed consistency with the present investigation, evaluated transportation and centering capabilities across five rotary systems in 2018, including ProTaper Gold, ProDesign S, Hyflex CM, Hyflex EDM, and ProDesign Logic. The research concluded that transportation remained similar across all systems, with ProDesign Logic and Hyflex EDM demonstrating superior performance [14]. Although the file systems evaluated by Pinheiro et al. differed from those used in the present study, their findings provide relevant contextual evidence regarding the shaping behavior of thermally treated NiTi systems and their ability to maintain canal curvature.

Lima et al. conducted a 2020 study demonstrating that Bassi Logic™ 0.03 system utilization during root canal preparation resulted in higher percentages of untouched canal surfaces compared to XP-endo Shaper and Reciproc instruments. No differences were observed between systems regarding dentin removal percentages [23]. This finding suggests that the superior centering ratio and reduced transportation observed with the Bassi Logic system may result from decreased file-canal contact and better preservation of original canal morphology.

The design characteristics of the Bassi Logic system, including its dedicated glide path instruments and thermally treated alloy, may contribute to more conservative canal shaping and preservation of the original canal anatomy. These features may partly explain the lower canal transportation observed in the present study.

The achievement of apical patency in 85% of molar teeth without manual file usage indicates that cervical pre-flaring becomes unnecessary in most cases. Without pre-flaring requirements, cervical dentin preservation occurs, maintaining more tooth structure to withstand applied stresses.

The taper and rectangular cross-section of glide path files prevent canal engagement and negotiation beyond the apical foramen [10]. Finishing files (featuring 0.03, 0.05, and 0.06 tapers) incorporate unique double helix cross-sections, variable blade spacing, and appropriate helical depth. These characteristics collectively reduce dentin extrusion and minimize dentin debris accumulation [11].

The characteristics of glide path files and the Bassi Logic finishing system indicate less invasive treatment approaches, reduced dentin removal, and apical foramen preservation. A 2014 study by Amr M., examining transportation, centering ratio, and remaining dentin thickness in two groups using ProTaper Next files, where one group utilized glide path files, concluded that the glide path group demonstrated significantly superior performance regarding transportation at 3 and 5 millimeters from the apex [12]. Therefore, the present study’s results may relate to glide path utilization in the Bassi Logic system, resulting in decreased transportation and maintained centering ratios.

It should also be noted that differences in glide path protocols between the two systems may have influenced canal transportation and centering outcomes. The Bassi Logic system incorporates a dedicated glide path instrument as part of its manufacturer-recommended sequence, whereas ProTaper Next preparation was preceded by manual glide path establishment using a #15 K-file. Therefore, the findings should be interpreted within the context of system-specific preparation protocols rather than isolated instrument geometry alone.

In addition, the two systems were operated at different rotational speeds according to their manufacturers’ recommendations. Although this approach reflects routine clinical use, rotational speed may influence cutting efficiency, file flexibility, debris removal, and canal transportation. Therefore, this factor should be considered when interpreting the comparative shaping performance of the two systems.

Apical transportation exceeding 0.3 millimeters can compromise apical seal integrity and treatment prognosis [7]. Although significant differences were observed between the two groups at two apical distances in this investigation, transportation measurements ranged between 0.065 and 0.145 millimeters, which does not compromise apical seal effectiveness. Therefore, both file systems demonstrate negligible transportation levels in terms of apical seal preservation.

Clinically, minimizing canal transportation is important because preservation of the original canal anatomy contributes to improved apical sealing, more predictable obturation, and reduced risk of procedural errors such as ledging, zipping, or apical deviation. Therefore, even small differences in transportation may be relevant when preparing curved canals, particularly in the apical third.

The present study has several limitations. First, this was an in vitro study; therefore, the findings may not fully reproduce clinical conditions. Second, periodontal ligament simulation was not performed, which may affect the external validity of the findings. Third, although CBCT provides non-destructive three-dimensional assessment, voxel-size limitations may affect the precision of very small linear measurements. Fourth, the sample size was relatively limited and restricted to mandibular first molars with 10–30° curvature; therefore, the results may not be directly generalizable to other tooth types or canals with severe curvature. Fifth, differences in glide path protocols and rotational speed between the two systems may have influenced canal transportation and centering outcomes. Finally, dedicated image registration software was not used for automatic superimposition of pre- and post-instrumentation CBCT images, although standardized positioning, reference grooves, orientation markers, and identical acquisition parameters were used to improve reproducibility.

## Conclusions

Within the limitations of this in vitro study, both rotary systems maintained canal transportation within clinically acceptable limits. However, ProTaper Next showed significantly greater canal transportation at 3 mm in the mesiodistal plane and at 5 mm in the buccolingual plane compared with Bassi Logic. No statistically significant difference was observed in centering ability between the two systems. These findings suggest that Bassi Logic may provide more conservative shaping at certain apical levels, although the results should be interpreted within the context of the different system-specific preparation protocols.

## Data Availability

The datasets used and/or analyzed during the current study are available from the corresponding author upon reasonable request.

## Abbreviations

NiTi: Nickel-Titanium
PTN: ProTaper Next
CBCT: Cone-Beam Computed Tomography
FOV: Field of View
DICOM: Digital Imaging and Communications in Medicine
